# Histo-blood group antigens as receptors for rotavirus, new understanding on rotavirus epidemiology and vaccine strategy

**DOI:** 10.1038/emi.2017.30

**Published:** 2017-04-12

**Authors:** Xi Jiang, Yang Liu, Ming Tan

**Affiliations:** 1 Cincinnati Children's Hospital Medical Center, Department of Pediatrics, University of Cincinnati College of Medicine, Cincinnati, OH 45229, USA

**Keywords:** diarrhea, evolution, HBGA, host receptor, rotavirus, vaccine

## Abstract

The success of the two rotavirus (RV) vaccines (Rotarix and RotaTeq) in many countries endorses a live attenuated vaccine approach against RVs. However, the lower efficacies of both vaccines in many low- and middle-income countries indicate a need to improve the current RV vaccines. The recent discovery that RVs recognize histo-blood group antigens (HBGAs) as potential receptors has significantly advanced our understanding of RV diversity, evolution and epidemiology, providing important new insights into the performances of current RV vaccines in different populations and emphasizing a P-type-based vaccine approach. New understanding of RV diversity and evolution also raises a fundamental question about the ‘Jennerian' approach, which needs to be addressed for future development of live attenuated RV vaccines. Alternative approaches to develop safer and more cost-effective subunit vaccines against RVs are also discussed.

## INTRODUCTION

Rotaviruses (RVs) are the principal cause of severe diarrhea in children, responsible for ~200 000 deaths, 2.3 million hospitalizations and 24 million outpatient visits among children under five years of age worldwide each year.^1, 2, 3^ Since 2006, two RV vaccines, Rotarix and RotaTeq, have been licensed and widely used in many countries around the world.^4, 5, 6, 7, 8, 9, 10, 11, 12, 13^ Rotarix consists of a single, live G1P[8] human RV that has been attenuated by multiple *in vitro* passages. RotaTeq contains five, live reassortant RVs, including four bovine P[5] reassortants combined with a single human G type (G1–G4), plus another bovine reassortant with human P[8](G6P[8]). Following their implementation through national immunization programs, both vaccines have effectively reduced RV disease burden against severe cases among vaccinated children in many industrialized countries throughout North America and Europe.^14, 15^

However, neither vaccine shows satisfactory efficacy in low- and middle-income countries, particularly in Africa and Asia.^16, 17, 18^ Many factors could contribute to these lower efficacies, including interference by high maternal antibody titers in infants, breastfeeding, concurrent infection with other microbes, micronutrient deficiencies and altered gut microbiomes. This indicates that children in the developing world are possibly different from those in developed countries,^19, 20, 21, 22^ but evidence supporting these hypotheses remains limited. A recent study showed that immune responses against Rotarix were not enhanced after withholding breastfeeding around the time of vaccination,^23^ which warrants further study to understand the low efficacies of the two, live RV vaccines in low- and middle-income settings.

According to the G-type-based ‘Jennerian' vaccine approach, the pentavalent RotaTeq should have a higher efficacy than the monovalent Rotarix, however, this is not observed. In fact, the monovalent Rotarix demonstrates similar or better protection than the pentavalent RotaTeq in both developed and developing countries. This scenario indicates a knowledge gap in our current understanding of RV epidemiology and host immunity as related to protection against RVs, which must be filled to develop more effective RV vaccines.

**Figure 1:**
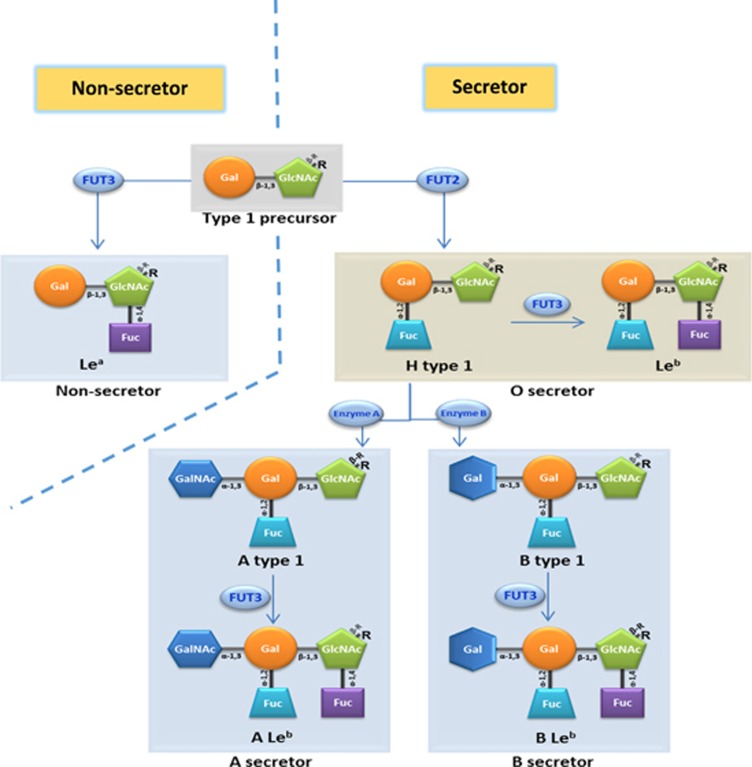
Schematic biosynthesis pathways of human type 1 histo-blood group antigens (HBGAs). Starting with the type 1 precursor (Galβ1-3GlcNAcβ-R), a Le^a^ antigen is formed by adding a Fuc to β-GlcNAc via a α-1,4 linkage by α-1,3/4-fucosyltransferase (FUT3). A Fuc can also be added to the β-Gal of the precursor via a α-1,2 linkage, forming H antigen by α-1,2-fucosyltransferase (FUT2). The H antigen can be further extended by adding another Fuc to β-GlcNAc via α-1,4 linkage to form a Le^b^ antigen via FUT3. Under the action of an *N*-acetylgalactosamine transferase (A enzyme), a GalNAc is added to the β-Gal of the H antigen via a α-1,3 linkage, forming an A antigen that can be further developed into an ALe^b^ antigen by accepting a α-1,4 Fuc through FUT3. Similarly, through galactosyltransferase (B enzyme), the β-Gal of the H antigen accepts a α-1,3 Gal, forming a B antigen that can develop into the BLe^b^ antigen. Individuals with the H, Le^b^, A, B, ALe^b^ and/or BLe^b^ antigens are secretors. A and/or B secretors may also carry small amount of H and Le^b^ antigens as intermediates. In contrast, individuals without an active FUT2 do not synthesize H antigen and the downstream A, B, A/B, ALe^b^ and/or BLe^b^ antigens are non-secretors. The syntheses of the type 2 HBGAs follow similar pathways starting with the type 2 precursor (Galβ1-4GlcNAcβ-R), resulting in Le^x^, H type 2, Le^y^, A type 2, ALe^y^, B type 2 and/or BLe^y^ antigens. l-fucose (Fuc); d-galactose (Gal); *N*-acetylgalactosamine (GalNAc); *N*-acetylglucosamine (GlcNAc); backbone of HBGAs (R).

The recent discovery that RVs recognize histo-blood group antigens (HBGAs) as attachment factors or receptors^24, 25, 26, 27, 28^ has significantly advanced our understanding of RV epidemiology, which may help to fill this knowledge gap. HBGAs are ubiquitous among world populations and are polymorphic with different ABO, Lewis and secretor versus non-secretor types (Figure 1). RVs are diverse in recognizing the polymorphic HBGAs in a strain-specific manner, which may contribute to strain-specific RV host ranges among different populations. In addition, HBGAs undergo stepwise biosynthesis by adding a saccharide during each step (Figure 1), a process that is developmentally regulated in children and could possibly lead to age-specific susceptibility to RV infection during their early lives.^29^ Furthermore, similar polymorphic HBGAs are also observed in many animals, depending on their glycosyltransferase makeups for ABO, H and Lewis families, resulting in some shared HBGA products with humans. These shared HBGAs may be responsible for RV cross-species transmission between humans and animals, further complicating RV epidemiology, disease burden and vaccine strategies.

Significant advancements have been made in elucidating complicated RV–host interactions over the past four to five years, which provide important new insights into the performance of the two current RV vaccines among different world populations in various geographic regions. These new advancements also raise questions about our traditional views of RV–host interaction, host range, host specificity and epidemiology, particularly zoonotic transmission of RVs between humans and animals, providing new information and alternative approaches, including non-replicating RV vaccines, for future vaccine development.

### HBGAs as important host factors or receptors for RVs

**Figure 2:**
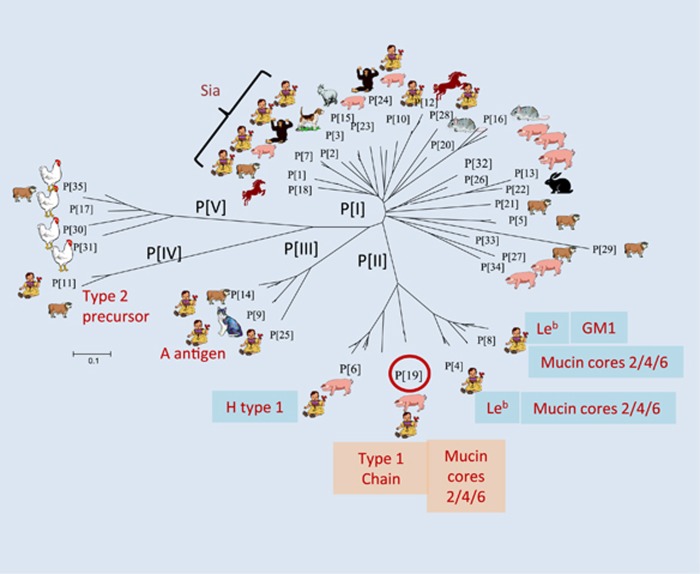
RVs recognize histo-blood group antigens (HBGAs). A total of 35 RV genotypes (P[1]–P[35]) in five genogroups (P[I]–P[V]) based on the VP8* sequences of the viral capsid structural protein VP4 have been classified following phylogenetic analysis.^34^ The potential target hosts for individual genotypes are indicated based on the frequencies of sequences found for individual genotypes in each species. The carbohydrate ligands or receptors for individual RV genotypes are based on recent publications,^34, 35, 36, 37^ but such data for most animal RVs remain unknown. The finding that RV Wa recognizes an internal SA is not indicated in the figure. The horizontal bar indicates the number of substitutions per amino acid. Sialic acid dependent (Sia).

Early studies showed that some animal RVs recognize terminal sialic acids (SAs),^30^ but subsequent studies found that most animal RVs and almost all human RVs are sialidase-insensitive,^31, 32^ and therefore SA-independent, although one human RV (strain Wa) has been found to recognize an internal SA.^33^ It was recently found that almost all P genotypes in genogroups P[II]–P[IV], that commonly infect humans, recognize HBGAs (Figure 2).^24, 25, 26, 27, 28, 34^ This has led to a plausible hypothesis that HBGAs are important host factors or cellular receptors for RVs.

The specific interactions between RVs and HBGAs have been demonstrated by *in vitro* binding of recombinant RV surface spike protein VP8* with the A, B, H (secretor) and Lewis antigens (Figure 2) from different sources, including human saliva, milk, synthetic oligosaccharides and red blood cells (hemagglutination). Glycan array analyses have also confirmed these RV–HBGA interactions. Specific virus–host interactions have also been demonstrated by resolving the atomic structures of VP8* proteins for select human RVs (P[14] and P[11]) in complex with their HBGA oligosaccharide ligands with X-ray crystallography ^28, 38^ and STD NMR analyses (P[19]).^39^ The associations between RV infection and a child's secretor status and Lewis types have also been observed through epidemiology and literature data studies,^24, 26, 27, 40, 41, 42^ strongly suggesting that HBGAs play an important role in RV infection, host range and pathogenesis.

### HBGAs are highly polymorphic with wide, global human distributions and are present in some animals

HBGAs are fucose-containing carbohydrates that are abundantly distributed on intestinal mucosal epithelia, where they serve as attachment factors or receptors for a variety of enteric viral pathogens, including RVs and noroviruses. HBGAs are synthesized from various disaccharide precursors, the major determinants of HBGA types 1–6, through consecutive additions of monosaccharides with specific linkages. The addition of each monosaccharide is catalyzed by a specific glycosyltransferase. Four major glycosyltransferases, the α-1,2 fucosyltransferase (FUT2), the α-1,3 or α-1,4 fucosyltransferase (FUT3), as well as the A and B enzymes (Figure 1), that are coded by three major HBGA gene families and involved in HBGA synthesis, resulting in ABO (A, B and/or H antigens), Lewis (Lewis a, b, x and/or y antigens) and secretor/nonsecretor (with/without H epitope) histo-blood types.

The human HBGAs are polymorphic with silent alleles in each glycosyltransferase, resulting in variable HBGA products for the ABO, H and Lewis families,^43, 44^ each with varying distributions among the world's populations. For example, H-positive individuals who contain an active FUT2 enzyme represent the majority (~80%) of European and North American populations and are the reason behind the predominance of certain RV P genotypes that recognize the H antigens. On the other hand, the Lewis-positive phenotype is controlled by the FUT3 enzyme, present in ~90% of the general population, but this rate is much lower in Africa, resulting in a higher rate of Le^a−b−^ (Lewis-negative) phenotype in this region,^45, 46, 47^ which may explain the higher prevalence of P[6] RVs in Africa compared to the rest of the world (see below).

The stepwise HBGA biosynthesis process is developmentally regulated in the early lives of children, leading to age-specific HBGA products, and thus age-specific RV host ranges. In addition, HBGA genetic polymorphism may occur in some animal species, leading to shared HBGAs between humans and animals, which may be the basis for the observed RV cross-species transmission. Furthermore, RVs are found to recognize mucin cores of mucin *O*-glycans,^39^ which can be further extended and branched with saccharide residues Gal, GlcNAc, Fuc or sialic acid that are commonly seen in HBGAs,^48^ further complicating RV diversity, host ranges and epidemiology, while impacting RV vaccine strategies.

### The major human RVs P[4], P[6] and P[8] recognize the type 1 HBGAs

The P[4], P[6] and P[8] RVs in the P[II] genogroup (Figure 2) cause over 95% of RV gastroenteritis cases in children worldwide.^49^ The recombinant VP8* domain in the VP4 spike protein of these three genotypes bound Le^b^ and/or H-type 1 antigens in oligosaccharide-based binding assays.^35^ These observations were further confirmed by saliva-binding assays, in which the binding signals for P[4] and P[8] VP8* to human adult saliva correlated with Le^b^ antigen expression (‘secretor'-positive individuals).^35^ The specificities of HBGA–RV interactions have also been demonstrated through the binding of authentic triple layer RVs, but not the double layer particles, further supporting the importance of the VP4/VP8* surface spike protein in host interaction and RV infection.^35^

P[6] RVs are genetically related to P[4] and P[8],^34, 35^ but mainly infect children in low- and middle-income countries.^50, 51, 52^ In addition, P[6] RVs are commonly found in animals (porcine), neonates and young infants; where infected neonates and young infants may be asymptomatic.^53, 54, 55^ Interestingly, the P[6] VP8* did not bind adult saliva, but interacted with neonate and young infants' saliva,^39^ and type 1 HBGA chains with or without the H epitope, including type 1 HBGA precursors without further terminal Lewis fucose modifications,^35, 39^ leading to the hypothesis that similar to P[11] RVs, P[6] has an age-specific host range and cross-species transmission between humans and animals (see below).

### HBGAs as potential factors for RV cross-species transmission

The P[9], P[14] and P[25] RVs in the P[III] genogroup are commonly detected in both humans and a variety of wild and domestic animals. For example, P[9] RVs are frequently detected in cats and dogs, while P[14] RVs often infect sheep, cattle and goats.^56, 57, 58, 59, 60^ These three genotypes bound to A antigens in oligosaccharide-binding assays,^28, 34^ while their recombinant VP8*s also bound human and animal mucins with a positive correlation between the binding signals and A antigens.^34^ Co-crystallization of the P[14] RV VP8* and A antigen confirmed the GalNAc residue (the A epitope) as the major determinant in P[14] RV–HBGA binding.^28^ These data strongly suggest that the shared A antigen between humans and these animal species could play a role in the observed cross-species transmission of P[III] genogroup RVs.

### Age-specific host ranges for P[11] and P[6] RVs

P[11] RVs commonly infect neonates and young infants, usually without symptoms.^61, 62, 63^ The P[11] VP8* was found to recognize tandem disaccharide repeats of Galβ1-4GlcNAc (poly-LacNAc), a type 2 HBGA precursor, as a host ligand or receptor.^36, 37, 64^ Expression of such precursor molecules is believed to be developmentally regulated,^65^ suggesting an age-specific host range for P[11] RVs. Further evidence supporting this age-specific host range included the strong interaction between P[11] VP8* and saliva from neonates and young infants, but not from adults.^36^ A similar binding preference for neonatal/young infant saliva was also identified for P[6] RVs,^39^ thus, both P[6] and P[11] may recognize human HBGA precursors; where P[11] recognizes the type 2 chain precursors and P[6] recognizes the type 1 chain precursors. These binding specificities have been further demonstrated using a human milk shotgun glycan array with the recombinant P[6] and P[11] VP8* proteins,^64^ and by X-ray crystallography of the P[11] VP8* in complex with its ligands.^38^

Both P[6] and P[11] are more commonly found in developing countries than developed ones,^61, 66, 67, 68, 69, 70, 71^ particularly for P[6], which causes ~30% of RV cases in Africa.^50, 51, 52, 66, 67, 68^ One possible reason could be the close contact between children and domestic animals in rural areas of developing countries, since these animals may be infected with P[6] and P[11] RVs due to shared HBGA ligands between humans and animals.^36, 38, 64^ Another possible reason for the high prevalence of P[6] in Africa could be the higher rates of Le^a−b−^ (Lewis-negative) phenotype in African populations,^45, 46, 47^ which possibly leads to a longer age-window with high expression levels of the HBGA precursors, since a correlation between P[6] RV infection and the Lewis-negative phenotype in children has been observed.^26^

### Tracing RV evolution with a potential animal host origin

Extended studies of P[19] RVs, that are rarely found in humans but commonly in animals (porcine), have revealed a unique binding property for P[19] RVs to mucin core and type 1 HBGA precursors as the functional units. Additional interactions with other residues, such as type 1 HBGA A, B, H and Lewis epitopes, also occur, which determine host ranges.^39^ Similar properties were also observed for all P[II] RVs, P[4], P[6] and P[8], indicating typical divergent evolution of the P[II] RVs selected by the polymorphic type 1 HBGAs.^39^ This hypothesis is supported by the finding of a common binding site on the VP8* surfaces of P[19], a feature that is shared with all three P[II] genotypes, as well as two animal RVs (P[10] and P[12]) in P[I], and supported by almost identical saliva- and glycan array-binding profiles for P[10] and P[19].^39^ P[10] was found in bats,^72^ but occasionally infects humans similar to P[19]. Thus, it is deduced that these animal and human RVs may represent a unique evolutionary lineage starting from a common ancestor in P[I] with a possible animal host origin. While the original binding property to mucin core and type 1 HBGA precursors is maintained among members of this evolutionary lineage, additional interactions with adjacent residues may have occurred when the ancestor was introduced into humans. This led to the diverse strains seen today, with some mainly infecting animals while others mainly infecting humans.^39^

### New understanding of RV epidemiology

The elucidation of P[II] RV evolution sheds light on RV epidemiology. For example, P[19] RVs may represent an early stage of RV evolution and recognize less abundant precursor disaccharides with limited additional HBGA residues, explaining why P[19] is rarely found in humans. On the other hand, the P[4] and P[8] RVs are more adapted and recognize more mature type 1 HBGAs, such as the Lewis b antigens that contain both the secretor and Lewis epitopes representing 80% and 90% of the general populations, respectively. Their adaptation and recognition explain the predominance of P[4] and P[8] in humans. In addition, the ability to bind additional residues, particularly the Lewis epitopes required for P[4] and P[8],^39^ explains why neither genotype is commonly found in animals, as the Lewis antigens are not commonly expressed in animals, such as mice^73^ and pigs (Jiang, 2015). However, P[8] RVs could be detected in domestic pigs,^74, 75, 76^ complicating the role of Lewis epitope in P[8] RV host ranges and species barriers between humans and animals; future studies are necessary to clarify this issue. Furthermore, the P[6] RVs may represent an RV evolutionary intermediate by adapting to limited glycan residues, such as the H epitope (α1–2 fucose). This could explain their prevalence in neonates and young infants, as well as in domestic animals, due to the premature HBGA products produced during certain ages that may also be shared with some animals. Finally, the P[10] and P[12] RVs in P[I] may represent an earlier cluster that retained the most animal host specificity, explaining why they are commonly found in animals, but rarely in humans.

Similar principles of evolution and epidemiology may also apply to other RV genotypes and genogroups. For example, the P[III] genogroup may diverge in parallel with P[II] under selection of the A antigens and develop a new binding site recognizing the GalNAc (the A epitope)-containing saccharides of A antigens as major functional units. The A antigen phenotypes are found in ~30% of the general population, explaining why P[III] RVs are significantly less common in humans than P[4] and P[8]. A antigen is also present in some animals, consistent with the common infection of many domestic and wild animals by P[III] RVs.^56, 57, 58, 59, 60^ Similarly, the P[IV] and P[V] genogroups could follow the divergent evolution path and gain new host ranges by changing their binding interface to fit new hosts. For example, the P[IV] RVs may be selected by type 2 HBGAs and mainly infect neonates, young infants and some animals by recognizing age-specific precursors shared with some animals, a scenario similar to P[6] RVs that recognize type 1 HBGA precursors. On the other hand, P[V] RVs represent an evolutionary lineage that may recognize an as-yet-unknown ligand unique to avian and bovine species that may be responsible for their susceptibility, which is not present in humans.

Finally, the large number of different genotypes in P[I] could also follow the same principle of divergent evolution from the common ancestor. Since the majority exclusively infect animals (Figure 2), they may recognize receptor ligands unique to individual animal species that are not shared with humans. On the other hand, a number of other P[I] genotypes, P[1]–P[3], P[7], P[24] and P[28], infect both animals and humans, and may recognize host receptors shared by humans and animals, such as the type 1 HBGA precursors recognized by P[10]. Since these cross-species reactive genotypes are potential zoonotic sources for human disease, future studies are important to determine their receptor ligands and worldwide distributions. It is noted that significantly high rates of untypeable P types were reported during RV surveillance in different countries, particularly under-developed African and Asian countries,^77, 78, 79, 80, 81^ leaving a knowledge gap on the true prevalence of these genotypes in human populations. Thus, efforts to improve typing methods for broad detection and include additional P types are important to facilitate our understanding of RV epidemiology and disease burden to develop better vaccine strategies against RVs.

### New insights into current RV vaccines and vaccine strategies

The new understanding of HBGA-controlled RV host ranges, evolution and epidemiology emphasizes the importance of the VP4/VP8* spike protein in RV infection and pathogenesis. This helps to address basic questions about the variable effectiveness of the two current RV vaccines (Rotarix and RotaTeq) among different populations in various geographic regions of the world. For example, although the G type makeups of the two vaccines differ significantly, both vaccines contain a common human P type (P[8]), which may explain why both vaccines are highly effective in many developed countries, since P[8] RVs are the most prevalent genotype worldwide. In addition, P[8]-based vaccines may also protect against P[4] RVs, as P[4] is genetically closely related to P[8] and recognizes common HBGA receptors.^82, 83^ On the other hand, P[8]-based vaccines may not protect against other P types, such as P[6] and P[11] RVs that are more prevalent in developing countries, possibly explaining the low efficacies of both vaccines in many African and Asian countries. Specific protection of children by VP4-specific neutralizing antibodies against natural RV infection has been clearly demonstrated.^84, 85^ Thus, the P[8]-based vaccine strains in both Rotarix and RotaTeq may play an important role in protection of vaccinated children. A cocktail vaccine containing major P types may broaden protection of children against RVs in both developed and developing countries.

The elucidation of RV host ranges and evolution also raises a fundamental question about the ‘Jennerian' approach for developing live, attenuated RV vaccines, which may further explain the effectiveness issues with the two current RV vaccines in certain populations. For example, RotaTeq is a pentavalent vaccine containing four bovine RV (P[5]) reassortants, each representing a major human G type (G1–G4). Since the P[5] RVs have been found to exclusively infect bovine, concern has been raised regarding their ability to replicate in vaccinated children. RotaTeq has been found to replicate poorly in vaccinated children, even at higher vaccine dosages, even poorer than the monovalent Rotarix containing only one vaccine strain.^86^ Thus, while the P[8]-based vaccine strains may replicate efficiently in human intestines due to matched host factors or receptors, the four P[5]-based RotaTeq reassortants may not replicate or replicate poorly in vaccinated children because they lack host factors for the bovine strains. This could explain why RotaTeq did not have higher efficacy than Rotarix. Thus, future studies are urgently important to determine whether the P[5] RV vaccine strains in RotaTeq replicate in vaccinated children, as the ‘Jennerian' approach using bovine P[5] and other animal P types is still widely used for RV vaccine development in many countries.^87, 88, 89, 90, 91, 92, 93, 94, 95, 96, 97^

The explanation that current RV vaccine efficacies are the result of their included P types supports the variable protection by different RV G types observed in many countries. For example, many RV G types, including G1, G3, G4, G8 and G9, are highly prevalent in humans but they frequently share a common P[8] type, explaining why the monovalent Rotarix, containing only one G1 type, is highly effective in many countries against different G types. In fact, the P[8] vaccine strain of RotaTeq carries the bovine G6 that is not commonly found in humans, thus, the observed ‘heterotypic protections' among different G types could be due to the common P[8] of the two current RV vaccines. On the other hand, a unique type-specific protection of RotaTeq against G8 RVs has been observed in African children.^98^ The G8 RVs are commonly coupled with P[6], which may not be cross-reactive with P[8]. Thus, a potential cross protection between G8 and G6 could explain the efficacy of RotaTeq against G8 RVs, as G8 and G6 are genetically closely related. Finally, while future studies are required, the deduced G6/G8 cross protection may also explain the heterologous protection of African children given RotaTeq against P[6] RVs.^99^

### Alternative approaches for future vaccine development against RVs

Given the new understanding about the current live, attenuated RV vaccines and their risk for intussusception in vaccinated children,^100, 101, 102^ alternative approaches with non-replicating RV vaccines may be considered for future vaccine development. Virus-like particle (VLP)-based vaccines containing different combinations of RV VP2/6/4/7 structural proteins have been studied, but none resulted in satisfactory effectiveness in mice and/or gnotobiotic pig models. As a result, a current recommendation for vaccine design would combine live attenuated and VLP vaccines to prime, then boost, respectively, immune responses and possibly avoid adverse effects.^103^

According to the new knowledge about the role of VP4/VP8* in RV–host interactions and infection following the discovery of HBGAs as potential RV receptors, a novel approach using non-replicating VP8*-based subunit vaccines has been proposed. One such VP8*-based subunit vaccine candidate induced significant levels of neutralizing antibodies in immunized mice.^104, 105^ In addition, a chimeric vaccine, containing VP8* and the tetanus toxoid universal CD4(+) T-cell epitope P2, induces higher titers of neutralizing antibodies than vaccines without the P2 epitope in immunized guinea pigs.^106^ The chimeric vaccine also significantly delayed the onset of diarrhea and reduced the duration and severity of diarrhea in gnotobiotic pigs.^106^

**Figure 3:**
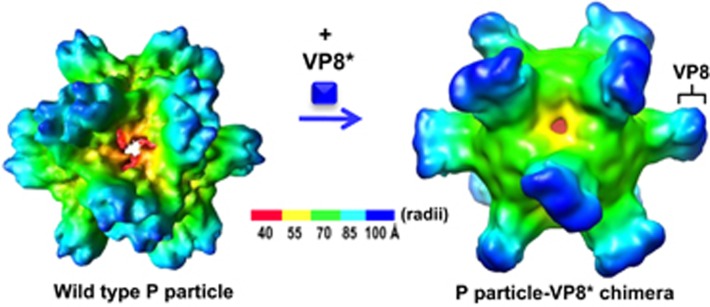
Cryo-EM structures of the norovirus (NoV) P particle (left) and the chimeric NoV P particle with a rotavirus (RV) surface spike protein VP8* (right). Each chimeric P particle contains 24 copies of the P-VP8* chimeric proteins forming 12 dimers with the VP8* presented on the surface of the P particle.

Efforts have also been made to enhance the immunogenicity of VP8* by increasing its valence via a polyvalent vaccine platform.^107^ A chimeric VP8* vaccine candidate containing 24 copies of the VP8* antigen has been made using the norovirus P particle as the carrier^108, 109, 110^ (Figure 3). Animal trials with this chimeric vaccine revealed significantly increased titers of VP8*-specific antibodies compared to those induced by the free VP8* in both ELISA-based assays, as well as cell culture-based neutralization tests.^111, 112, 113^ Finally, the chimeric VP8* vaccine also exhibited significantly higher levels of protection against RV infection in a mouse model.^111^ Based on the same principle, chimeric VP8* vaccines displayed in polyvalent complexes, the lineage and network polymers,^114, 115^ have also been made and exhibited significantly increased immunogenicity against VP8* in mice and increased neutralizing activity against RV replication in cell culture.^114, 115, 116^ In summary, the recombinant VP8*-based subunit vaccine candidates are promising alternatives for future vaccines against RVs.

## CONCLUSION AND FUTURE PERSPECTIVE

The recent discovery that RVs recognize HBGAs as potential host receptors has significantly improved our understanding of RV diversity, host ranges and evolution, highlighting the role of the VP4/VP8* protein in RV infection and pathogenesis. New understanding of HBGA-controlled RV host ranges in different populations, including age-specific susceptibility of children to RV infection, significantly improved our knowledge of RV epidemiology. This provides valuable new insights into the performance of current RV vaccines (Rotarix and RotaTeq) in different populations, emphasizing the P-type-based vaccine approach. The elucidation of HBGA-controlled host ranges also sheds light on RV cross-species transmission between humans and animals, providing new theories regarding the current RV vaccines and the ‘Jennerian' vaccine approach, which is important for future RV vaccine development using the live, attenuated reassortants. The new information about RV diversity and evolution also indicates the necessity for continual RV surveillance in different populations and variable geographic regions with distinct social and economic statuses, particularly those in developing countries and remote areas. Improved methods for broad detection and accurate typing are also important to better understand RV epidemiology and select the ideal strains/genotypes to create broadly effective vaccines against RVs. The increased risk of intussusception reported in vaccinated children with both of the current RV vaccines suggests that non-replicating vaccine approaches are reasonable choices for future RV vaccine development. As a result, the currently developed VP8*-based subunit vaccines are promising alternative candidates. Technology to improve the productivity and immunogenicity of these VP8*-based vaccine candidates has been demonstrated following the display of VP8* antigens in various large, polyvalent protein complexes. Applications of these new technologies have made the VP8*-based vaccines promising candidates for the next generation of RV vaccines. Finally, while significant advancements have been made, the hypothesis that HBGA genetics play a major role in vaccine response remains to be validated in large cohort studies.
